# Vitexin suppresses the proliferation, angiogenesis and stemness of endometrial cancer through the PI3K/AKT pathway

**DOI:** 10.1080/13880209.2023.2190774

**Published:** 2023-03-30

**Authors:** Cuixia Liang, Yongjie Jiang, Lizhu Sun

**Affiliations:** a Department of Gynecology, The Fourth Affiliated Hospital, Zhejiang University School of Medicine, Yiwu, China; b Department of Gynecology and Obstetrics, Zheng Zhou Big Bridge Hospital, Zhengzhou, China; c Department of Oncology, Shuyang Hospital, The Affiliated Shuyang Hospital of Xuzhou Medical University, Suqian, China

**Keywords:** HEC-1B, Ishikawa, 740Y-P, Ki-67, PCNA, VEGFA, FGF2, OCT4, Nanog, *Vitex negundo*

## Abstract

**Context:**

Endometrial cancer is a common gynecologic malignancy. Vitexin is an active flavonoid compound with an antitumor function.

**Objective:**

This study elucidated the role of vitexin in endometrial cancer development and clarified the potential mechanism.

**Materials and methods:**

The toxicity of vitexin (0–80 μM) treatment for 24 h on HEC-1B and Ishikawa cells was tested utilizing the CCK-8 assay. Endometrial cancer cells were divided into vitexin 0, 5, 10, and 20 μM groups. Cell proliferation, angiogenesis and stemness *in vitro* after treatment with vitexin (0, 5, 10, 20 μM) for 24 h were evaluated using the EdU staining assay, tube formation assay and sphere formation assay, respectively. Twelve BALB/c mice were grouped into control and vitexin (80 mg/kg) groups to monitor tumour growth for 30 days.

**Results:**

Vitexin suppressed cell viability of HEC-1B (IC_50_ = 9.89 μM) and Ishikawa (IC_50_ = 12.35 μM) cells. The proliferation (55.3% and 80% for HEC-1B; 44.7% and 75% for Ishikawa), angiogenesis (54.3% and 78.4% for HEC-1B; 47.1% and 68.2% for Ishikawa) and stemness capacity (57.2% and 87.3% for HEC-1B; 53.4% and 78.4% for Ishikawa) of endometrial cancer cells were inhibited by 10 and 20 μM vitexin. Furthermore, the inhibitory effects of vitexin on endometrial cancer were reversed by PI3K/AKT agonist 740Y-P (20 μM). Moreover, the xenograft tumour experiment lasting for 30 days proved that vitexin (80 mg/kg) blocked tumour growth of endometrial cancer *in vivo*.

**Discussion and conclusions:**

Vitexin has therapeutic potential on endometrial cancer, which supports further clinical trials.

## Introduction

Endometrial cancer is a frequent gynaecologic malignancy derived from the endometrial lining of the uterus and approximately 75–85% of cases are adenocarcinoma (type I endometrial cancer) (Emons and Gründker 2021; Crosbie et al. 2022). Endometrial cancer ranks fourth among the death causes of gynaecological cancer worldwide (Wu et al. 2021). The morbidity and mortality are gradually increasing (Ferlay et al. 2015). It is estimated that the morbidity of endometrial cancer globally will increase by over 50% in 2040 compared with 2018 (Bray et al. 2018; Wu et al. 2021). For early-stage endometrial cancer patients, the 5-year overall survival is over 75% (Burke et al. 1990; Wang et al. 2019). However, the prognosis is still poor due to the loss of opportunity for surgery and effective chemotherapy drugs for endometrial cancer patients with metastasis (Burke et al. 1990; Wang et al. 2019). Therefore, exploring effective therapeutic strategies for endometrial cancer is crucial.

Vitexin (apigenin-8-C-d-glucopyranoside) is an active flavonoid compound that exists in the leaves and seeds of *Vitex negundo* Linn. (Lamiaceae) and a broad range of medicinal plants (Peng et al. 2021; Huang et al. 2022). This compound exerts antioxidative, anti-inflammatory and analgesic functions in some diseases (Borghi et al. 2013; Wei et al. 2014; Ożarowski and Karpiński 2021). In recent years, increasing evidence reveals the antitumor role of vitexin in cancer development (He et al. 2016; Liu et al. 2019; Ma et al. 2021; Huang et al. 2022). For example, Huang et al. (2022) discovered that vitexin blocked the aggressive behaviours of glioblastoma cancer cells and promoted apoptosis *via* modulating the JAK/STAT3 pathway. Liu et al. (2019) showed that vitexin accelerated non-small cell lung cancer cell apoptosis through PI3K/AKT/mTOR signalling. He et al. (2016) reported that vitexin promoted apoptosis in hepatocellular carcinoma *via* activating the JNK pathway. Vitexin effectively restrained the proliferation and inhibited the cell cycle and tumour growth in ovarian cancer (Ma et al. 2021). Nevertheless, the function of vitexin on endometrial cancer remains to be further elucidated.

The PI3K/AKT pathway is a vital pathway that regulates cell metabolism, proliferation, survival and angiogenesis (He et al. 2021). The PI3K/AKT pathway exerts a critical function on cancer development (He Y et al. 2021). Interestingly, the PI3K/AKT pathway often shows alterations and overactive in type I endometrial cancer (Lee and Kim 2014; Dong et al. 2019). Inhibiting the PI3K/AKT pathway is an effective treatment strategy for cancer (He et al. 2021). Wu et al. (2021) found that FABP4 suppressed the aggressive behaviours of endometrial cancer cells through modulating the PI3K/AKT pathway. Vitexin could regulate the PI3K/AKT pathway (Liu et al. 2019; Zhou et al. 2021). For instance, vitexin weakened the malignant development of gastric cancer *via* restraining activation of the PI3K/AKT/HIF-1α pathway (Zhou et al. 2021). Therefore, we inferred that vitexin might regulate endometrial cancer progression *via* modulation of the PI3K/AKT pathway.

Hence, this study elucidated the role of vitexin in endometrial cancer progression *via* determining cell proliferation, angiogenesis, and stemness and clarified the mechanism.

## Materials and methods

### Cell culture

Human normal endometrial stromal cells (HESCs) and human endometrial cancer cell lines HEC-1B and Ishikawa were acquired from BeNa Culture Collection (Beijing, China). Cells were grown in the DMEM-F12 medium plus 10% FBS at a 37 °C cell incubator with 5% CO_2_. At indicated experiments, cells were treated with different doses of vitexin (0, 1.25, 2.5, 5, 10, 20, 40 or 80 μM) [purity ≥ 95.0% (HPLC); Sigma, St. Louis, MO] or PI3K/AKT agonist 740Y-P (20 μM) (Wei et al. 2020) (Selleck Chemicals, Shanghai, China).

### Cell counting kit 8 (CCK-8) assay

Cells were aliquoted into 96-well plates (10^4^ cells/well). After cells were grown for 24, 48 and 72 h, 10 µL of CCK-8 was mixed to cells for 4 h incubation. Next, the optical density at 450 nm was recorded with a microplate reader (Multiskan MK3, Thermo Fisher Scientific, MA).

### EdU staining assay

Click-iT^®^ assay EdU Alexa Fluor^®^ 594 Imaging Kit (Thermo Scientific, Waltham, MA) was utilized to perform EdU staining. HEC-1B and Ishikawa cells were treated with 10 µM EdU labelling reagent for 2 h. After that, cells were immobilized in 4% polyformaldehyde (PFA) and incubated with 0.5% Triton X-100. Then, cells were dyed with Click-iT reaction cocktail without light. Cell nuclei were labelled with Hoechst 33342 without light. The EdU-positive cells were analysed utilizing a fluorescent microscope (LSM800, Zeiss, Gottingen, Germany).

### Western blot

Mouse tumor tissues and HEC-1B and Ishikawa cells were lysed utilizing RIPA reagent (Beyotime, Shanghai, China). Lysis samples were quantified using the BCA method, then resolved in SDS-PAGE and electro-transferring onto the PVDF membranes. Next, the membranes were impeded using 5% skim milk and treated with primary antibodies including anti-PCNA (1:1000; ab92552), anti-Ki-67 (1:5000; ab16667), anti-VEGFA (1:1000; ab51745), anti-FGF2 (1:1000; ab92337), anti-OCT4 (1:1000; ab200834), anti-Nanog (1:1000; ab21624), anti-p-PI3K (1:500; ab182651), anti-Total-PI3K (1:1000; ab133595), anti-p-AKT (S473) (1:500; ab8932), anti-p-AKT (T308) (1:500; ab38449), anti-Total-AKT (1:500; ab300473) and anti-β-actin (1:5000; ab8227) antibodies (Abcam, Cambridge, UK). After that, the membranes were treated with IgG H&L (HRP) (ab6721; Abcam, Cambridge, UK). Finally, the blots were measured utilizing the ECL chemiluminescence system and quantified using Image J software (NIH, Bethesda, MD).

### Tube formation assay

HEC-1B and Ishikawa cells were aliquoted in 6-well plates (1 × 10^5^ cells/well) and grown overnight in DMEM-F12 medium. The medium was then changed, and cells were maintained for 48 h with 2% FBS. The cell supernatants were collected and centrifuged for 5 min under 400 *g* as a conditioned medium. Then, a 96-well plate was pre-covered with Matrigel. HUVECs (3 × 10^4^ cells/well) were aliquoted into the 96-well plates and grown for 24 h using a conditioned medium. After that, the tube formation result was photographed utilizing a microscope and detected the total length of tubes per observation field using Image J software.

### Sphere formation assay

HEC-1B and Ishikawa cells were aliquoted into ultra-low attachment 6-well plates. The cells were maintained in the DMEM-F12 plus 2% B27, 20 ng/mL bFDF, 20 ng/mL EGF, 0.4% BSA and 5 μg/mL insulin for 14 days to culture the spheres (Ren et al. 2019). The spheres (>50 μm in diameter) were photographed and analysed under a light microscope (Axiostar Plus, Zeiss, Gottingen, Germany).

### Xenograft tumor experiment

Twelve BALB/c mice aged 6 weeks were acquired from Charles River Laboratories (Beijing, China). All mice were grouped into the control group and the Vitexin group. To construct the xenograft tumour, HEC-1B cells (1 × 10^7^) were injected subcutaneously into the mice. The mice in the Vitexin group were injected intraperitoneally with 80 mg/kg of vitexin twice weekly for 4 weeks (Zhao et al. 2020). An equal volume of normal saline was used to treat the control group. After 5 days of injection, tumour growth was recorded every 5 days until 30 days. Tumour volume was determined utilizing the formula: *V* = (Length × Width^2^)/2. Afterward, mice were euthanasia with CO_2_ inhalation, and tumours were excised to conduct Hematoxylin-Eosin (HE), Immunohistochemistry and Western blot assay. The research was authorized by the Ethics Committee of The Affiliated Shuyang Hospital of Xuzhou Medical University Shuyang People’s Hospital (2020-SYH-005) on 16 March 2020 and followed national and international regulations and policies.

### Immunohistochemistry and HE staining

Tumour tissues were fixed in 4% PFA, embedded using paraffin, and made into 5 µm slices. The slices were then deparaffinized, rehydrated, inactivated endogenous peroxidase and hindered non-specific binding. For immunohistochemistry, the slices were dyed with primary antibodies including Ki-67 (1:200), OCT4 (1:200) and VEGFA (1:200) (Abcam, Cambridge, UK) overnight, followed by treating with IgG H&L (HRP) (1:1000) (Abcam, Cambridge, UK) and staining with the DAB (R&D Systems, MN). For HE staining, the slides were treated with hematoxylin for 5 min, differentiated by 1% ethanol hydrochloride for 2 s and dyed with eosin for 2 min. The staining results were analysed under a microscope (LSM800, Zeiss, Gottingen, Germany).

### Statistical analysis

Data were represented by mean ± standard deviation (SD) and statistically analysed utilizing SPSS Statistics 22.0 (SPSS, Chicago, IL). All assays were conducted at least three times. Student’s *t*-test (non-paired) assessed the significance of the two groups. One-way ANOVA evaluated the significance among multiple groups with LSD’s *post hoc* test. *p* < 0.05 was the criterion of statistically significant.

## Results

### Vitexin suppresses endometrial cancer cell proliferation

**Figure 1.**
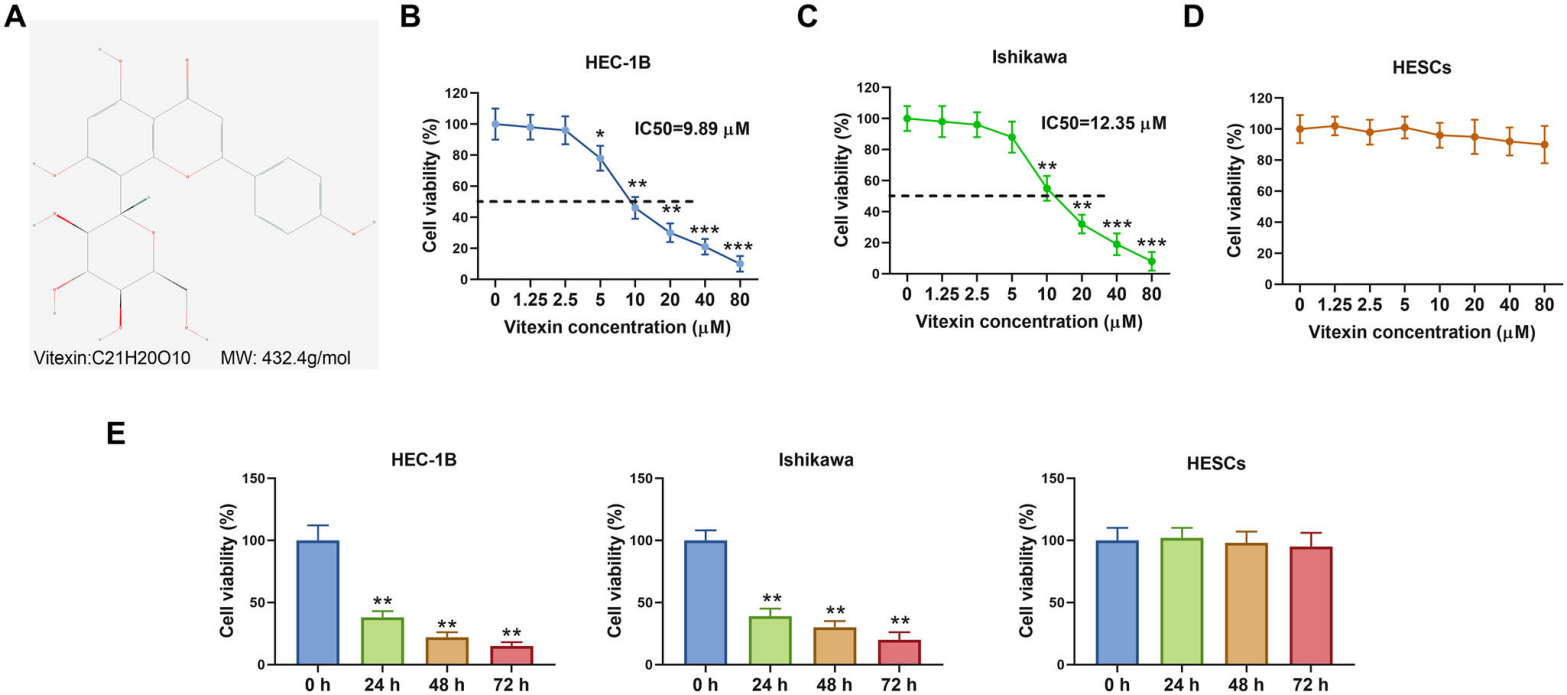
Vitexin suppresses endometrial cancer cell viability. (A) The chemical structure of vitexin. (B) CCK-8 assay measured the cell viability of HEC-1B after treatment with vitexin (0, 1.25, 2.5, 5, 10, 20, 40, 80 μM) for 24 h. (C) CCK-8 assay measured the cell viability of Ishikawa after treatment with vitexin (0, 1.25, 2.5, 5, 10, 20, 40, 80 μM) for 24 h. (D) CCK-8 assay measured the cell viability of HESCs after treatment with vitexin (0, 1.25, 2.5, 5, 10, 20, 40, 80 μM) for 24 h. (E) CCK-8 assay measured the cell viability of HEC-1B, Ishikawa and HESCs after treatment with 10 μM vitexin for 24, 48 and 72 h. *p < 0.05; **p < 0.01; ***p < 0.001 vs. vitexin 0 μM group or 0 h group.

**Figure 2.**
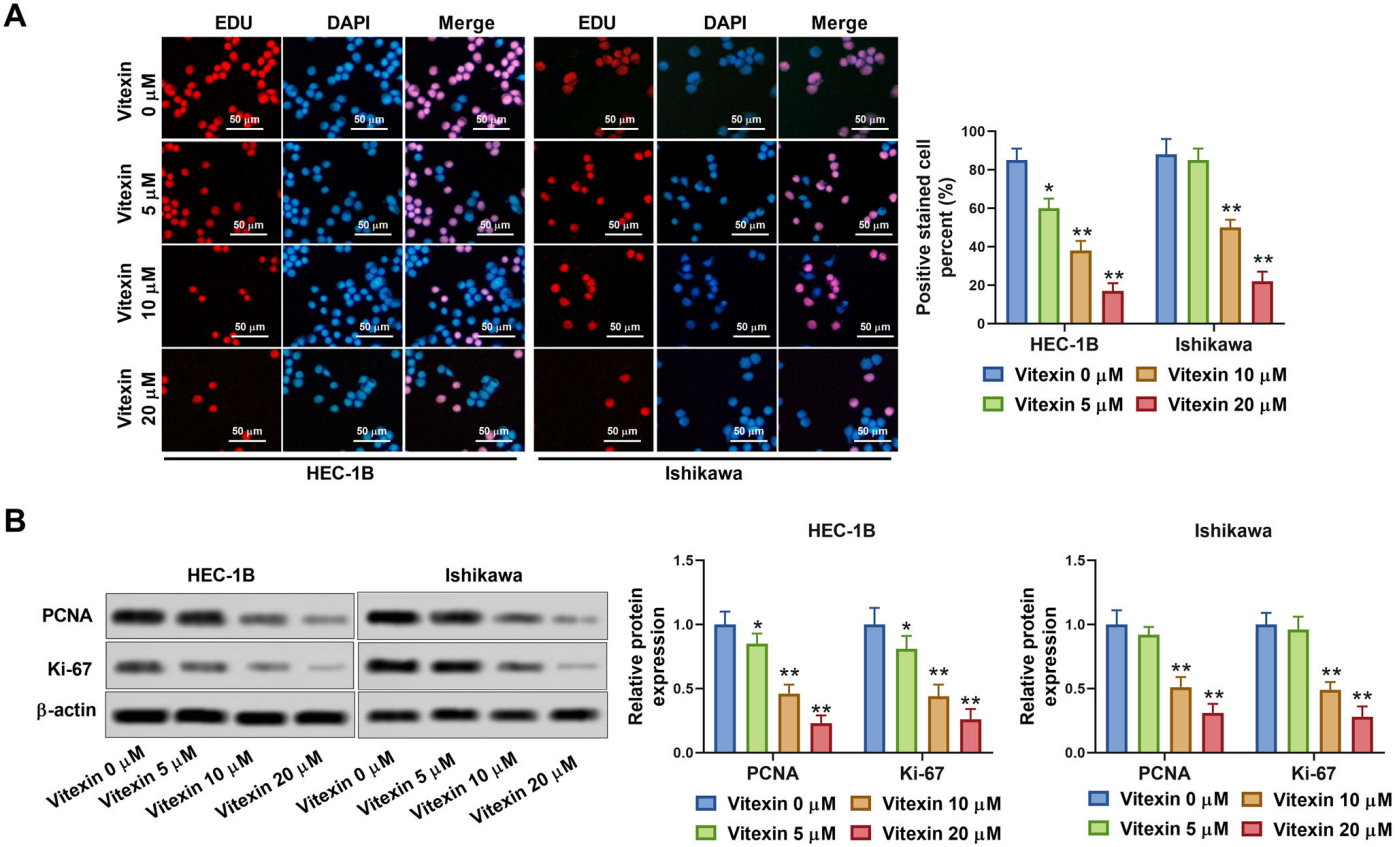
Vitexin suppresses endometrial cancer cell proliferation. (A) Cell proliferation of HEC-1B and Ishikawa after treatment with vitexin (0, 5, 10, 20 μM) for 24 h was assessed using EdU staining assay. (B) The protein levels of Ki-67 and PCNA after treatment with vitexin (0, 5, 10, 20 μM) for 24 h were measured using Western blots. *p < 0.05; **p < 0.01 vs. vitexin 0 μM group.

To clarify the action of vitexin on endometrial cancer development, endometrial cancer cells were stimulated with vitexin. The chemical structure of vitexin is presented in Figure 1(A). Results showed that 10, 20, 40 and 80 μM vitexin significantly inhibited cell viability of HEC-1B and Ishikawa, while 1.25 and 2.5 μM vitexin had no effect on cell viability (*p* < 0.05, Figure 1(B–C)). The IC_50_ of vitexin for inhibition of HEC-1B and Ishikawa cells was 9.89 μM and 12.35 μM, respectively. Given that 5, 10 and 20 μM vitexin exhibited higher inhibitory efficiency on cell viability, 5, 10 and 20 μM vitexin were selected for subsequent experiments. However, vitexin had no significant effect on cell viability of HESCs (Figure 1(D)). Besides, cell viability of HEC-1B and Ishikawa cells was significantly decreased after treated by 10 μM vitexin for 24, 48 and 72 h, respectively (*p* < 0.01, Figure 1(E)). Nevertheless, vitexin had no effect on HESCs viability at different treatment time (Figure 1(E)). Besides, vitexin inhibited cell proliferation of HEC-1B and Ishikawa, with an inhibition rate of over 50% for 10 and 20 μM vitexin (*p* < 0.05, Figure 2(A)). Furthermore, the levels of cell proliferation markers Ki-67 (0.85-, 0.46- and 0.23-fold change for HEC-1B cells and 0.92-, 0.51- and 0.31-fold change for Ishikawa cells in the 5, 10 and 20 μM vitexin groups, respectively) and PCNA (0.81-, 0.44- and 0.26-fold change for HEC-1B cells and 0.96-, 0.49- and 0.28-fold change for Ishikawa cells in the 5, 10 and 20 μM vitexin groups, respectively) were significantly decreased after vitexin treatment (*p* < 0.05, Figure 2(B)). Therefore, vitexin suppressed endometrial cancer cell proliferation.

### Vitexin suppresses endometrial cancer cell angiogenesis

**Figure 3.**
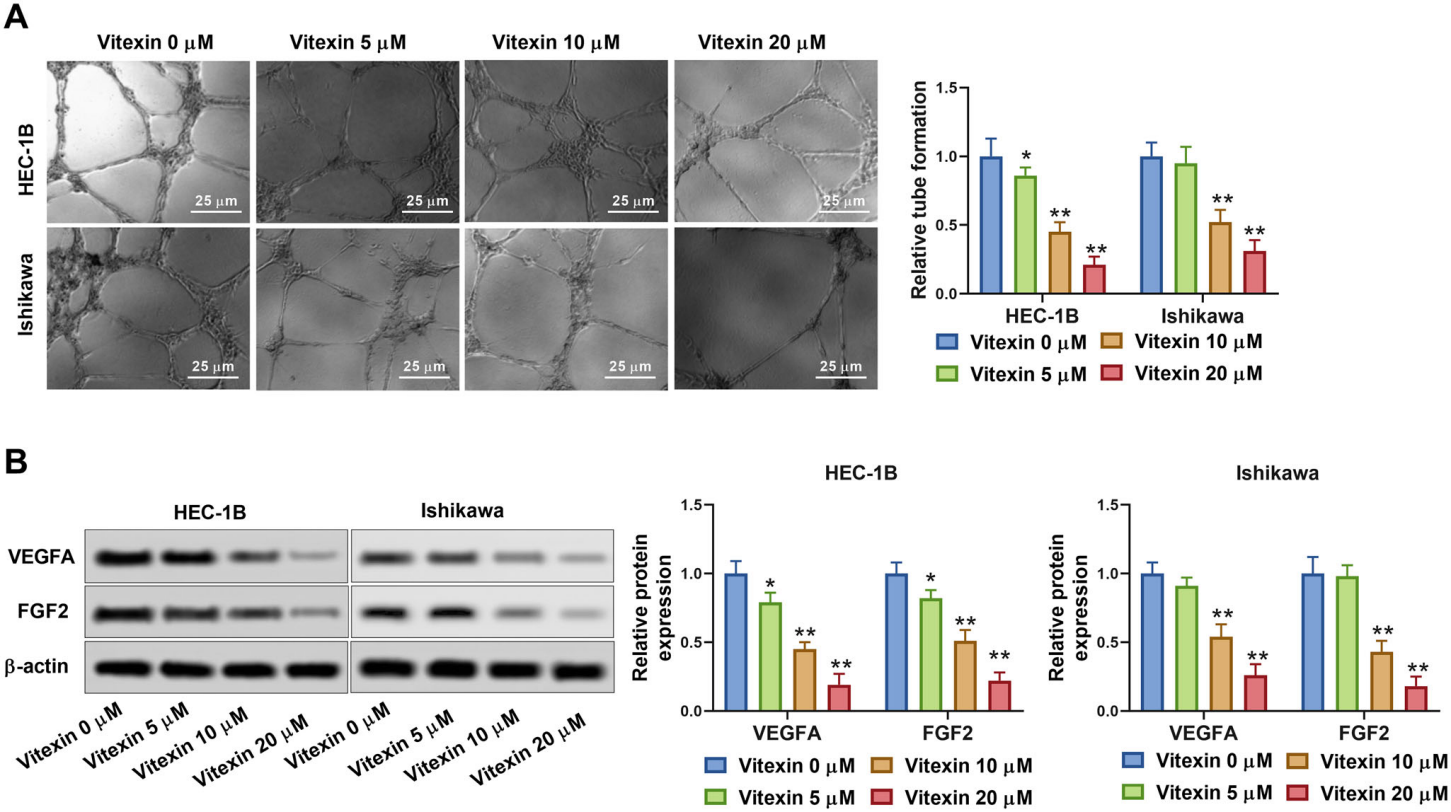
Vitexin suppresses endometrial cancer cell angiogenesis *in vitro*. HEC-1B and Ishikawa cells were treated with vitexin (0, 5, 10, 20 μM) for 24 h. (A) Angiogenesis ability of HEC-1B and Ishikawa cells was evaluated using the tube formation assay. (B) The protein levels of VEGFA and FGF2 were measured using Western blots. *p < 0.05; **p < 0.01 vs. vitexin 0 μM group.

To examine the role of vitexin in endometrial cancer progression, the tube formation capacity of endometrial cancer cells was assessed after treatment with 5, 10 or 20 μM vitexin. Vitexin considerably blocked the tube formation ability of HEC-1B and Ishikawa cells (*p* < 0.05, Figure 3(A)). Besides, the levels of angiogenic factors VEGFA (0.79-, 0.45- and 0.19-fold change for HEC-1B cells and 0.91-, 0.54- and 0.26-fold change for Ishikawa cells in the 5, 10 and 20 μM vitexin groups, respectively) and FGF2 (0.82-, 0.51- and 0.22-fold change for HEC-1B cells and 0.98-, 0.43- and 0.18-fold change for Ishikawa cells in the 5, 10 and 20 μM vitexin groups, respectively) were inhibited by vitexin (*p* < 0.05, Figure 3(B)). Thus, vitexin suppressed endometrial cancer cell angiogenesis.

### Vitexin suppresses endometrial cancer cell stemness capacity

**Figure 4.**
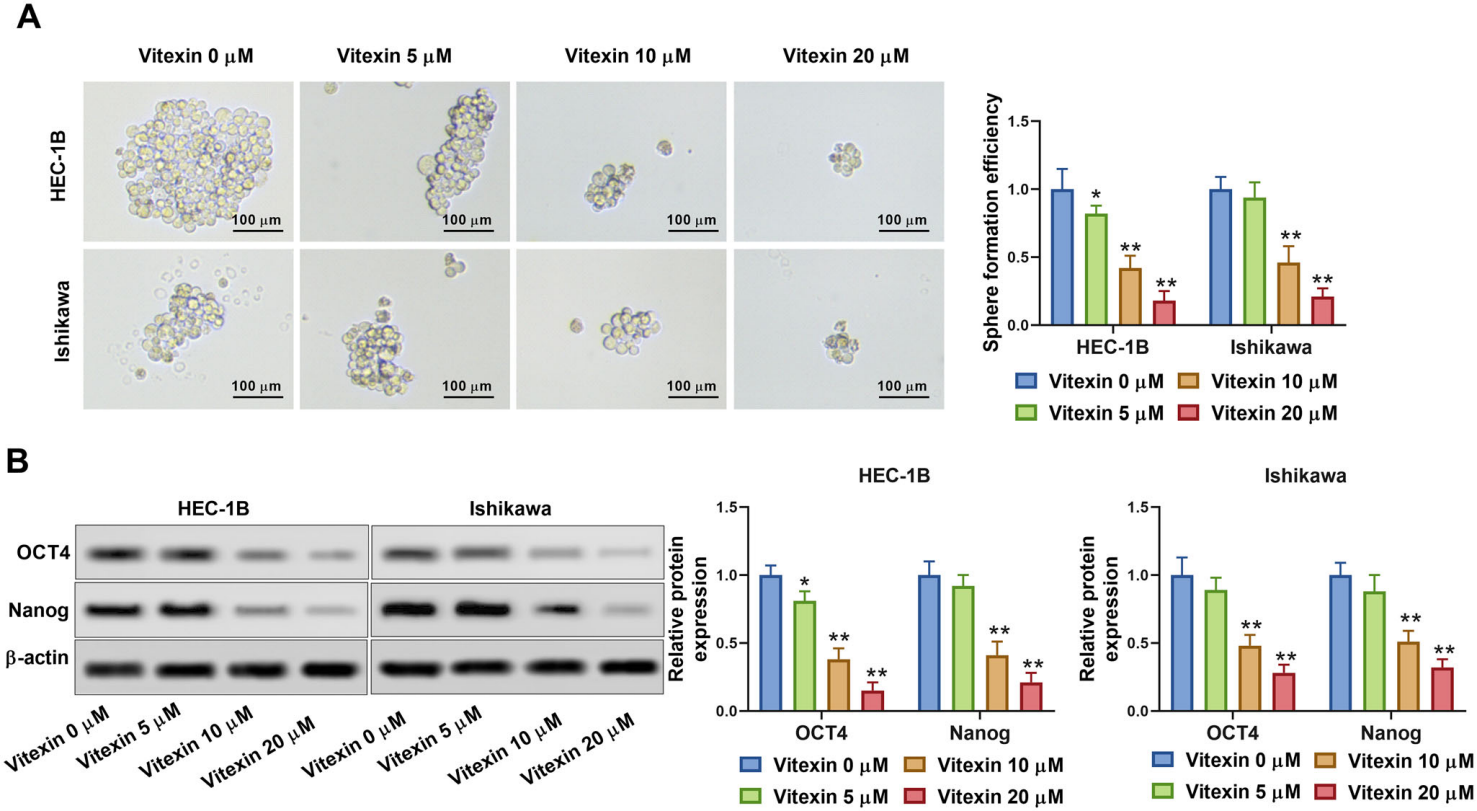
Vitexin suppresses endometrial cancer cell stemness capacity. HEC-1B and Ishikawa cells were treated with vitexin (0, 5, 10, 20 μM) for 24 h. (A) Stemness capacity of HEC-1B and Ishikawa cells was evaluated using sphere formation assay. (B) The protein levels of OCT4 and Nanog were measured using Western blots. *p < 0.05; **p < 0.01 vs. vitexin 0 μM group.

To better investigate the effect of vitexin on endometrial cancer aggressive behaviours, the stemness of endometrial cancer cells was assessed *via* sphere formation assay after treatment with vitexin. Results showed that vitexin retrained the sphere formation efficiency of HEC-1B and Ishikawa cells (*p* < 0.05, Figure 4A, Supplementary File 1). Furthermore, the stemness markers OCT4 and Nanog were suppressed by vitexin (*p* < 0.05, Figure 4(B)). Hence, vitexin restrained endometrial cancer cell stemness capacity.

### Vitexin suppresses the malignant phenotype of endometrial cancer via inhibiting the PI3K/AKT pathway

**Figure 5.**
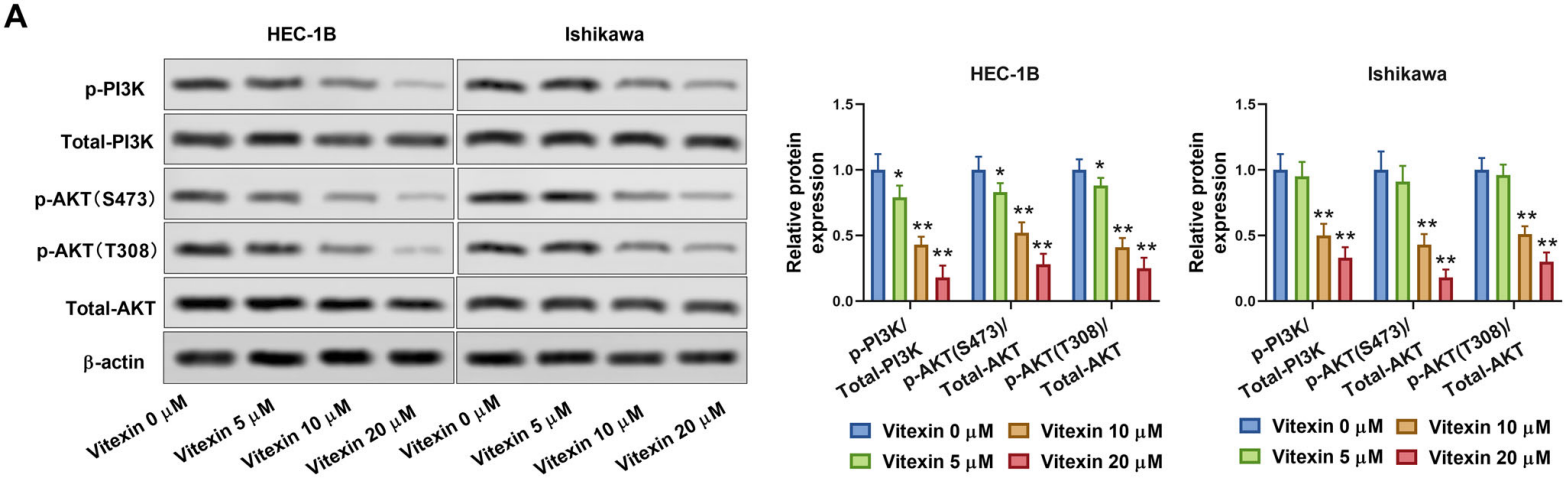
Vitexin suppresses activation of the PI3K/AKT pathway. (A) The protein levels of p-PI3K, total PI3K, p-AKT (S473), p-AKT (T308), and total AKT after treatment with vitexin (0, 5, 10, 20 μM) for 24 h were measured using Western blots. *p < 0.05; **p < 0.01 vs. vitexin 0 μM group.

**Figure 6.**
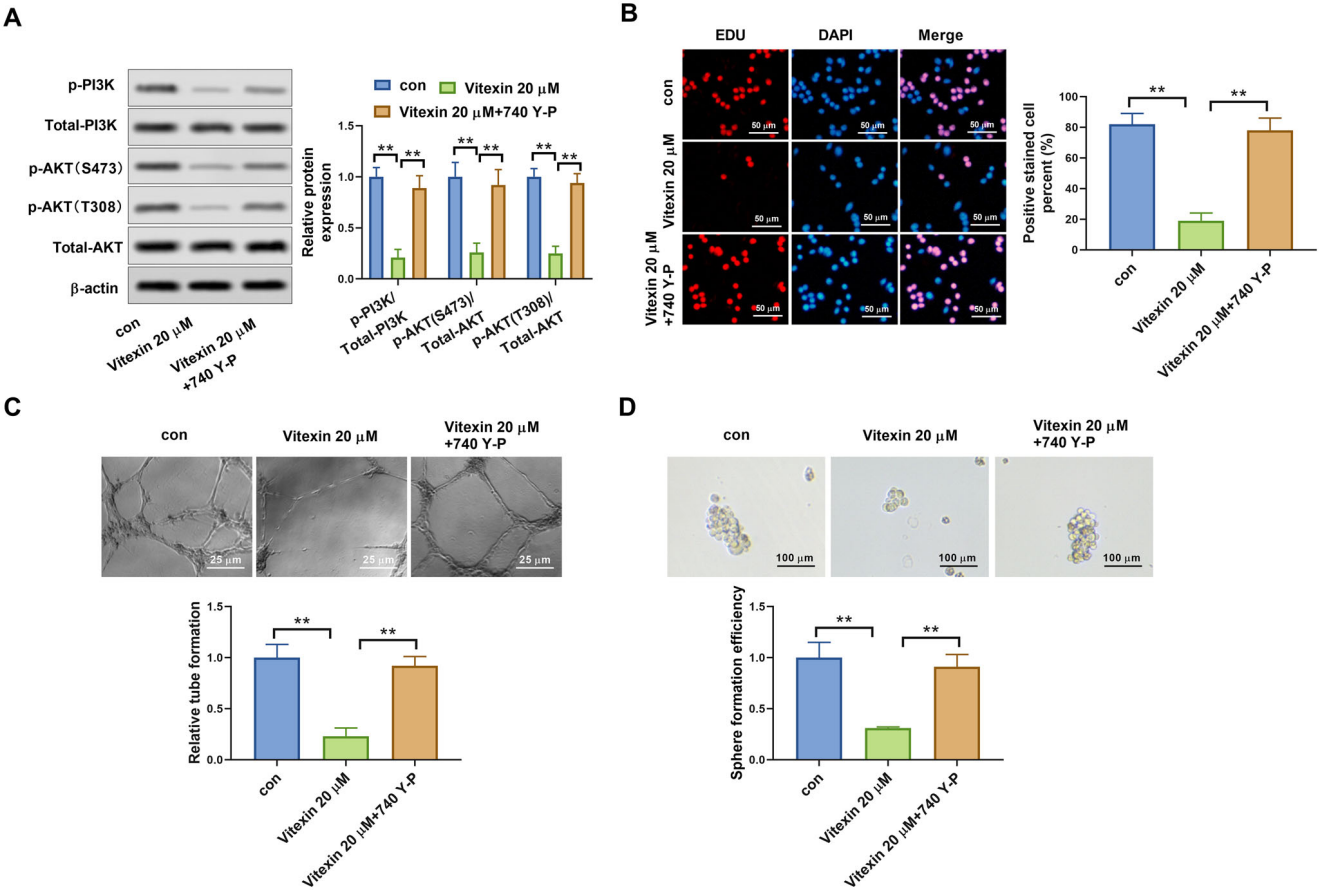
Vitexin suppresses the malignant phenotype of endometrial cancer *via* inhibiting the PI3K/AKT pathway. (A) The protein levels of p-PI3K, total PI3K, p-AKT (S473), p-AKT (T308), and total AKT after treatment with 20 μM vitexin and 20 μM 740Y-P for 24 h were measured using Western blots. (B) EdU staining assay determined the cell proliferation of HEC-1B cells after treatment with 20 μM vitexin and 20 μM 740Y-P for 24 h. (C) Tube formation assay assessed the angiogenesis ability of HEC-1B cells after treatment with 20 μM vitexin and 20 μM 740Y-P for 24 h. (D) Sphere formation assay evaluated the stemness capacity of HEC-1B after treatment with 20 μM vitexin and 20 μM 740Y-P for 24 h. **p < 0.01 vs. control group.

To illustrate the potential mechanism of vitexin on the regulation of endometrial cancer malignant phenotype, the PI3K/AKT pathway in endometrial cancer cells was determined after treatment with vitexin. Results revealed that vitexin treatment remarkably blocked the phosphorylation of PI3K and AKT but had no effect on total PI3K and AKT levels (*p* < 0.05, Figure 5A). PI3K/AKT agonist 740Y-P was then used to treat HEC-1B cells. The 740Y-P enhanced the phosphorylation levels of PI3K and AKT (*p* < 0.01, Figure 6(A)). In addition, EdU staining results found that vitexin suppressed the HEC-1B and Ishikawa cell proliferation, which was reversed after 740Y-P treatment (*p* < 0.01, Figure 6(B)). Furthermore, 740Y-P treatment abolished the inhibition influence of vitexin in the tube formation ability of HEC-1B cells (*p* < 0.01, Figure 6(C)). Moreover, the 740Y-P treatment also reversed the inhibition influence of vitexin in the stemness capacity of HEC-1B cells (*p* < 0.01, Figure 6(D)). Collectively, vitexin suppressed the malignant phenotype of endometrial cancer *via* inhibiting the PI3K/AKT pathway.

### Vitexin suppresses tumour growth of endometrial cancer *in vivo*

**Figure 7.**
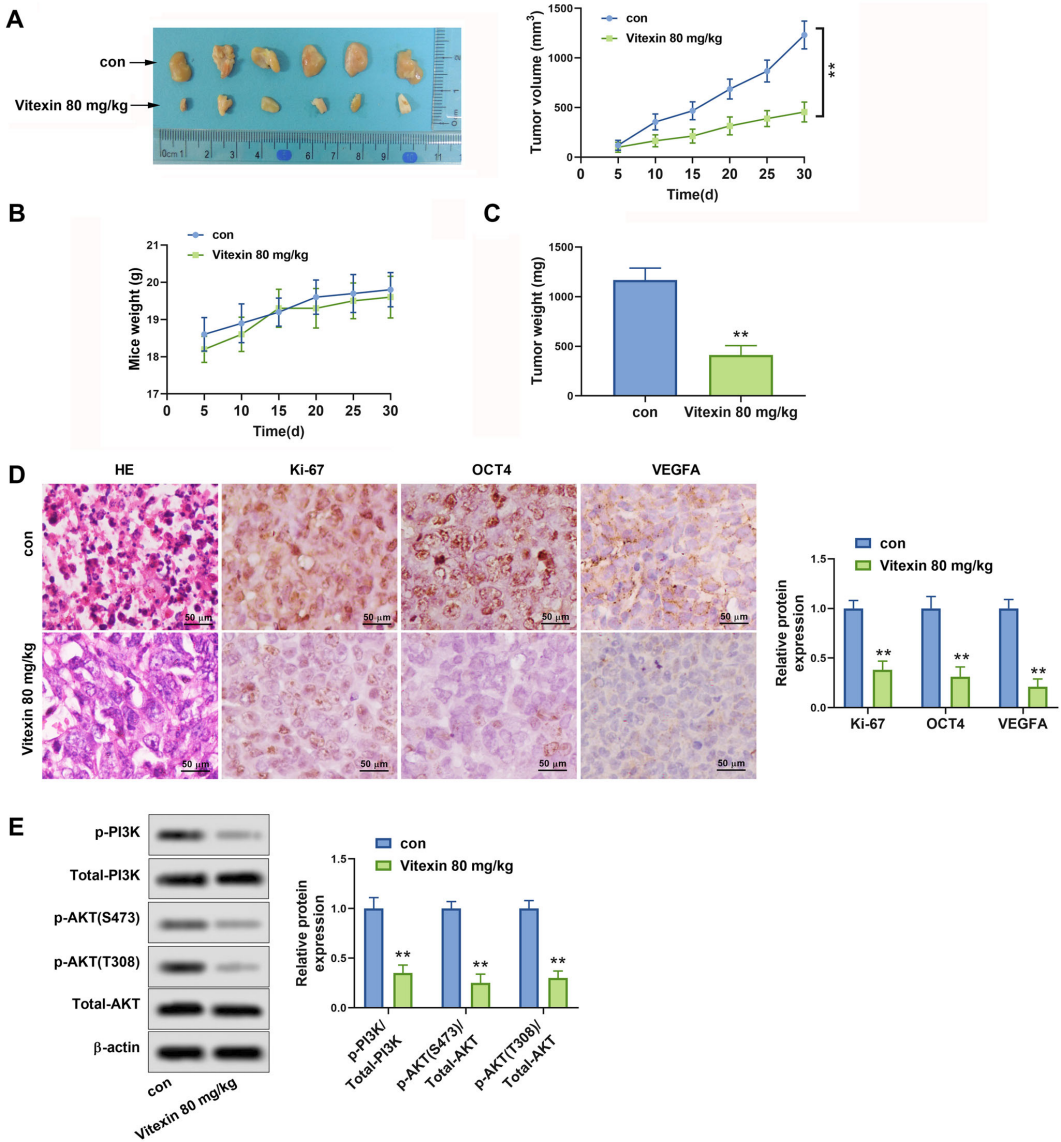
Vitexin suppresses tumour growth of endometrial cancer *in vivo*. HEC-1B cells (1 × 10^7^) were injected subcutaneously into mice of the control group and Vitexin group. Each group contained 6 mice. The mice in the Vitexin group were injected intraperitoneally with 80 mg/kg of vitexin twice weekly for 4 weeks. The mice in the control group were given the same amount of normal saline. (A) Images of the xenograft tumours from all mice at the endpoint and the tumour volumes of mice after treatment with vitexin. (B) The body weights of mice after treatment with vitexin. (C) The tumour weights of mice after treatment with vitexin. (D) The proliferation of tumour cells was evaluated by HE staining, and the levels of Ki-67, OCT4, and VEGFA in tumour tissues after treatment with vitexin were measured using Immunohistochemistry. (E) The protein levels of p-PI3K, total PI3K, p-AKT (S473), p-AKT (T308), and total AKT in tumour tissues after treatment with vitexin were determined using Western blots. **p < 0.01 vs. control group.

To interpret the role of vitexin in endometrial cancer progression *in vivo*, a xenograft tumour was constructed using HEC-1B cells. It was shown that vitexin restrained the tumour growth of endometrial cancer (*p* < 0.01, Figure 7(A–C)). Besides, HE results displayed that vitexin suppressed the proliferation of cancer cells (Figure 7(D)). Immunohistochemistry results revealed that the Ki-67, OCT4, and VEGFA were inhibited by vitexin in tumour tissues (*p* < 0.01, Figure 7(D)). The phosphorylation of PI3K and AKT were significantly reduced after treatment with vitexin in tissues (*p* < 0.01, Figure 7(E)). In general, vitexin suppresses tumour growth of endometrial cancer *in vivo*.

## Discussion

Endometrial cancer is a frequent gynaecologic malignancy with high incidence and poor prognosis (Ferlay et al. 2015; Emons and Gründker 2021; Wu et al. 2021; Crosbie et al. 2022). Accumulating evidence shows that vitexin plays an antitumor role in cancers (He J-D et al. 2016; Liu et al. 2019; Ma et al. 2021). Nevertheless, the action of vitexin on endometrial cancer is elusive. Hence, we studied the effect of vitexin on endometrial cancer using HEC1B and Ishikawa cells. HEC1B and Ishikawa cells are most used type I endometrial cancer-derived cell lines, harbouring alterations in the PI3K/AKT pathway (Van Nyen et al. 2018). Results demonstrated that vitexin repressed endometrial cancer cell proliferation. The finding was consistent with the previous studies (An et al. 2015; Ma et al. 2021; Huang et al. 2022). Huang et al. (2022) revealed that vitexin treatment restrained the proliferation of glioblastoma U251 cells. Ma et al. (2021) indicated that the proliferation and cell cycle of ovarian cancer cells were blocked by vitexin. An et al. (2015) proved that vitexin presented marked inhibitory roles in the proliferation of the esophageal cancer EC-109 cells. Ki-67 is a nuclear protein that is only produced in actively dividing cells (Sobecki et al. 2016; Kitson et al. 2017). Therefore, Ki-67 is considered to be a valuable marker of proliferation (Sobecki et al. 2016). PCNA, another marker of cell proliferation, is closely associated with the prognosis and survival in many malignant (Lu et al. 2019). This research found that the levels of Ki-67 and PCNA were remarkably inhibited by vitexin. The above evidence suggested that vitexin suppressed endometrial cancer cell proliferation.

Angiogenesis is a critical event in cancer development because tumour growth and metastasis depend on this process (Nishida et al. 2006). Blood supply is required if solid tumours grow beyond a minimum size of 2–3 mm^3^ (Abdelrahim et al. 2010). Interestingly, after tumours grow beyond the limitation of oxygen diffusion, hypoxia stimulates angiogenesis *via* hypoxia-inducible transcription factors (HIFs) (Abdelrahim et al. 2010). Therefore, inhibition of angiogenesis is considered an effective strategy to restrain tumour growth. In this study, tube formation assay demonstrated that vitexin treatment effectively suppressed endometrial cancer cell angiogenesis ability *in vitro*. Similarly, Wang et al. (2014) found that vitexin compound 1 suppressed angiogenesis of hepatocellular carcinoma. VEGFA is a major stimulator of angiogenesis, and it could induce endothelial cell proliferation and then directly lead to angiogenesis (Wang S et al. 2018). FGF2, another prototype angiogenic factor, is crucial for angiogenesis in both HAECs and HUVECs (Seo et al. 2016). Therefore, we determined the effect of vitexin on the levels of VEGFA and FGF2 and revealed that vitexin suppressed the level of VEGFA and FGF2. In brief, these findings proved that vitexin blocked the angiogenesis ability of endometrial cancer cells *in vitro*.

It has been accepted that cancer cell stemness is a cause of treatment failure and relapse in cancer cases (Ouban 2021). Cancer stem cells are identified in various cancers, exhibiting the self-renew capacity and proliferation ability like normal stem cells (Posada et al. 2017; Prasad et al. 2020). Therefore, the drug targeting cancer cell stemness is beneficial for cancer treatment. In this research, we studied the action of vitexin on the stemness capacity of endometrial cancer. It was revealed that vitexin retrained the sphere formation ability. Besides, the expression levels of OCT4 and Nanog were also inhibited by vitexin. OCT4 and Nanog are the stemness biomarkers, and they play crucial roles in maintaining stem cell pluripotency and proliferation ability (Zhang et al. 2013; Bai et al. 2015). Thus, vitexin suppressed the stemness capacity of endometrial cancer cells. Based on these data, we currently report the inhibitory influence of vitexin in stemness for the first time.

The PI3K/AKT pathway is an important signal pathway involved in proliferation, cell survival, and angiogenesis regulation in cancer cells, including endometrial cancer (He Y et al. 2021; Wu et al. 2021). Previous studies reported that the PI3K/AKT pathway mediated the antitumor effect of vitexin in cancers (Liu et al. 2019; Zhou et al. 2021). Therefore, we determined whether vitexin regulated endometrial cancer progression *via* the PI3K/AKT pathway. It was found that vitexin suppressed proliferation, angiogenesis, and stemness of endometrial cancer *via* restraining the activation of the PI3K/AKT pathway, which was consistent with the previous work (Liu et al. 2019; Zhou et al. 2021). Therefore, vitexin restrained the malignant phenotype of endometrial cancer *via* suppressing the PI3K/AKT pathway.

To verify the results of *in vitro* experiments, the xenograft tumour assay was carried out to elucidate the action of vitexin on endometrial cancer *in vivo*. As expected, vitexin suppressed tumour growth of endometrial cancer *in vivo*. Similarly, Zhou et al. (2021) showed that vitexin blocked the tumour growth of gastric cancer. Zhao et al. (2020) proved that vitexin significantly inhibited tumour growth of epithelial ovarian cancer *in vivo*. These findings may offer the potential for vitexin to become a promising therapeutic drug for endometrial cancer. However, it is unclear whether vitexin inhibits tumour growth *in vivo* of endometrial cancer *via* regulating the PI3K/AKT pathway. This is a limitation of this study and the next study will address the issue.

## Conclusions

These findings suggest vitexin has therapeutic potential on endometrial cancer progression *via* inactivation of the PI3K/AKT pathway. This study provides a theoretical basis for vitexin to be a valuable drug for endometrial cancer therapy, which needs further clinical trials to validate.

## Supplementary Material

Supplemental Material

## Data Availability

The datasets used and/or analyzed during the current study are available from the corresponding author on reasonable request.
