# Supplementary material for: Vitexin suppresses the proliferation, angiogenesis and stemness of endometrial cancer through the PI3K/AKT pathway
Source: Pharm Biol. 2023 Mar 30;61(1):581–9. doi: 10.1080/13880209.2023.2190774 (PMC10064825; doi:10.1080/13880209.2023.2190774)

Figure 3A-HEC-1B Vitexin 0 µM

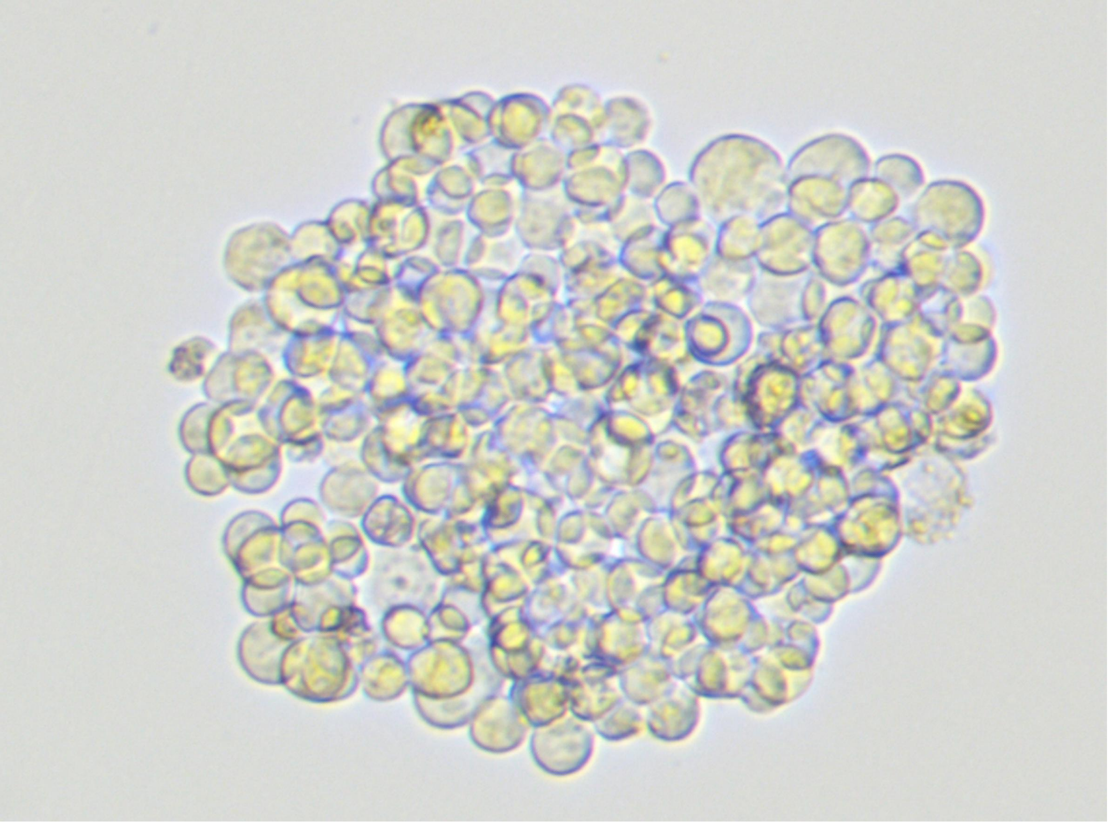

Figure 3A-HEC-1B Vitexin 5 µM

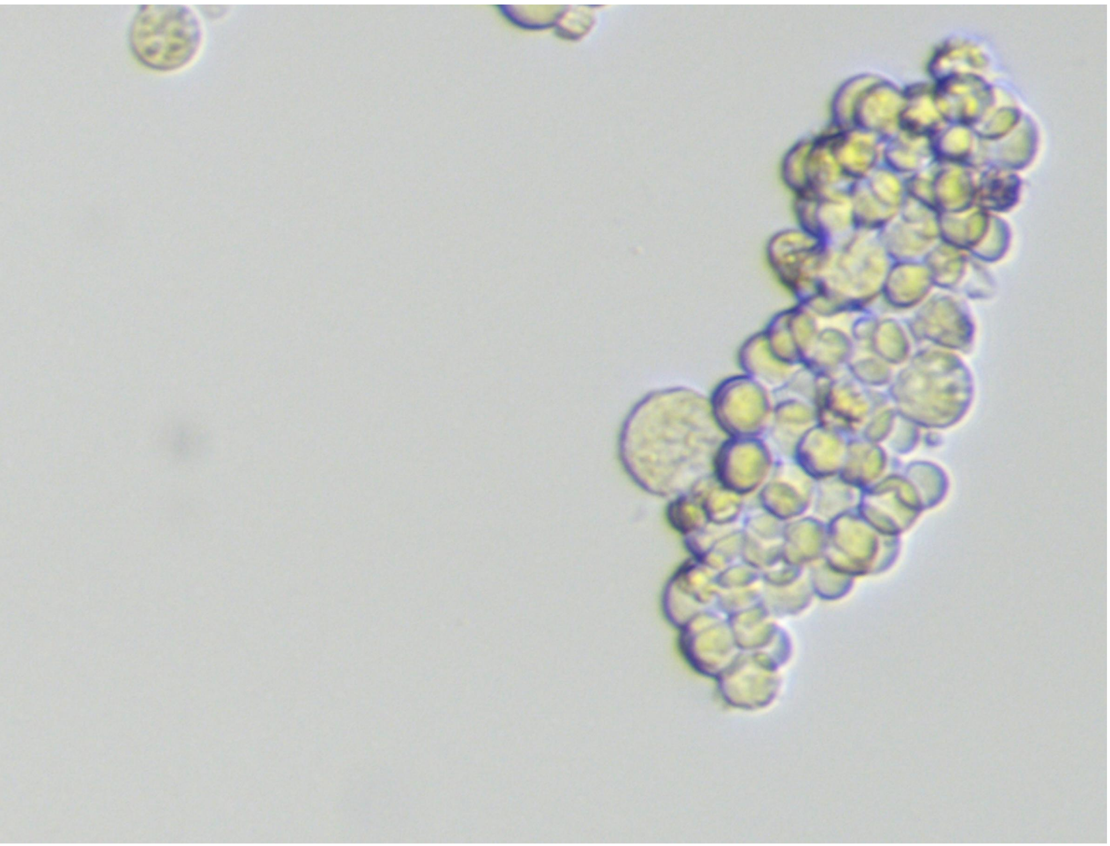

Figure 3A-HEC-1B Vitexin 10 µM

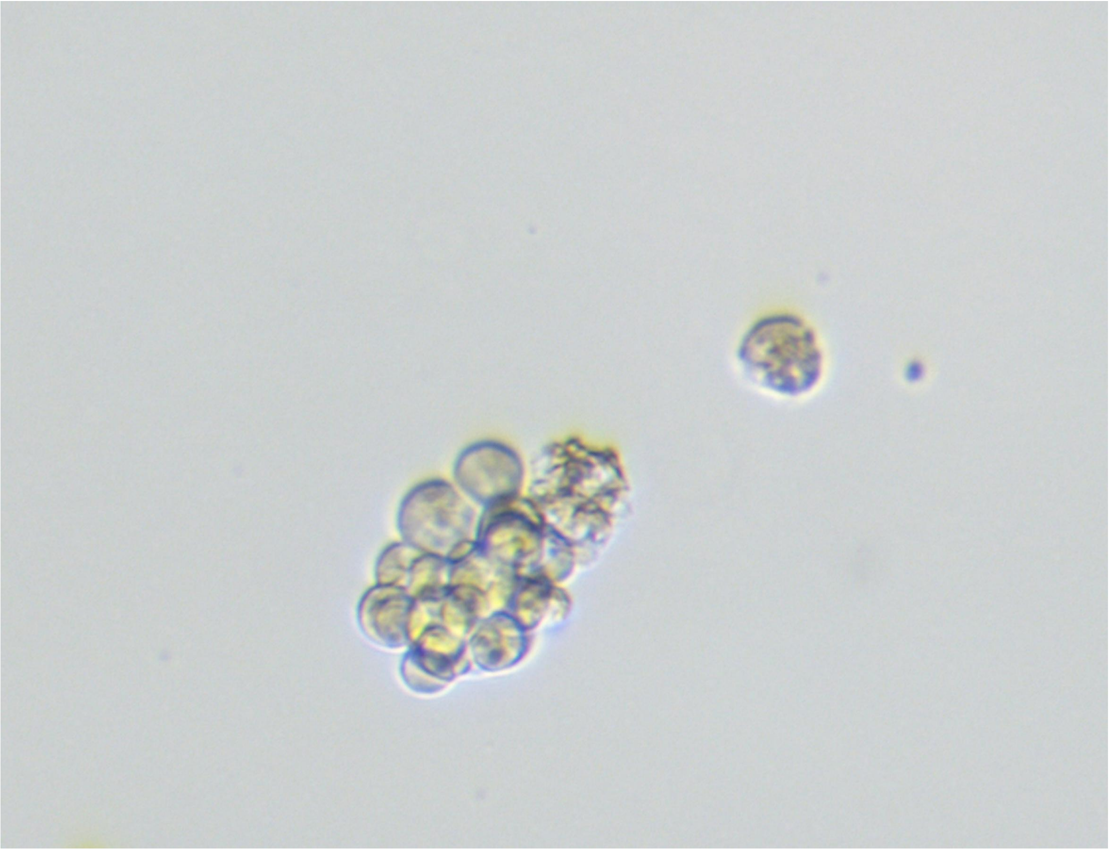

Figure 3A-HEC-1B Vitexin 20 µM

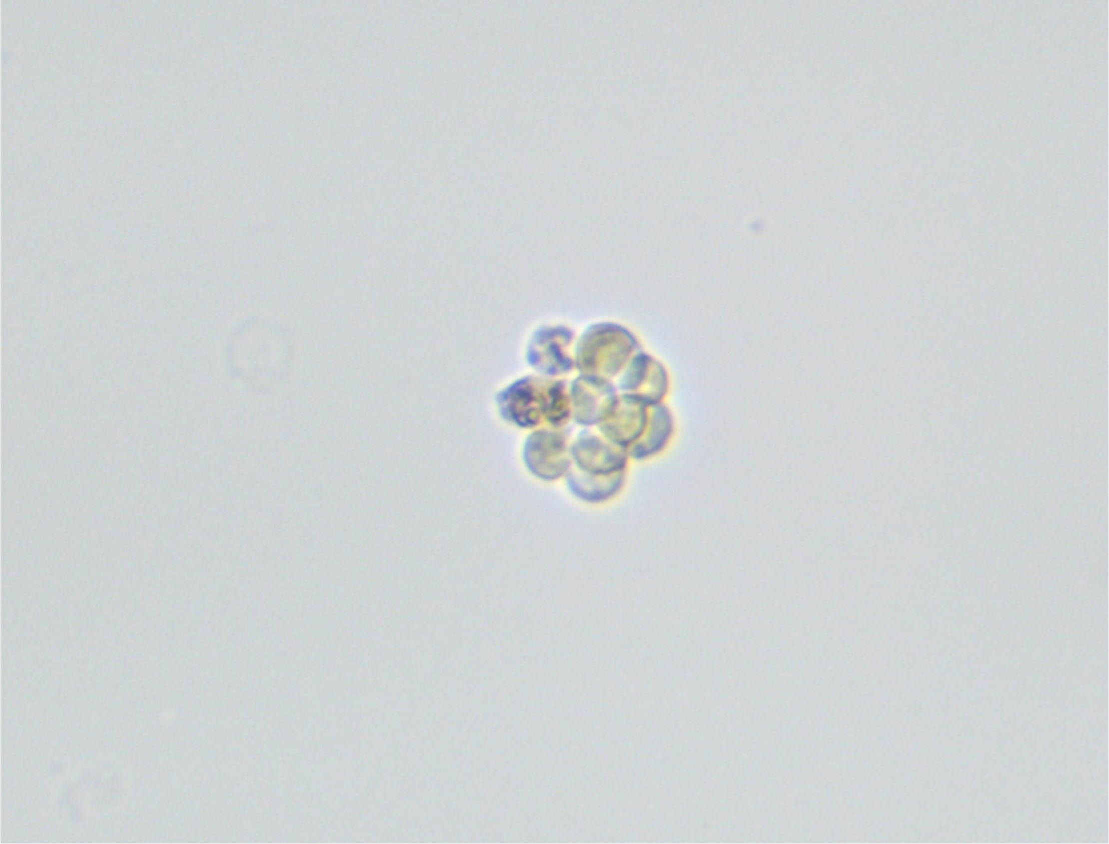

Figure 3A-Ishikawa Vitexin 0 µM

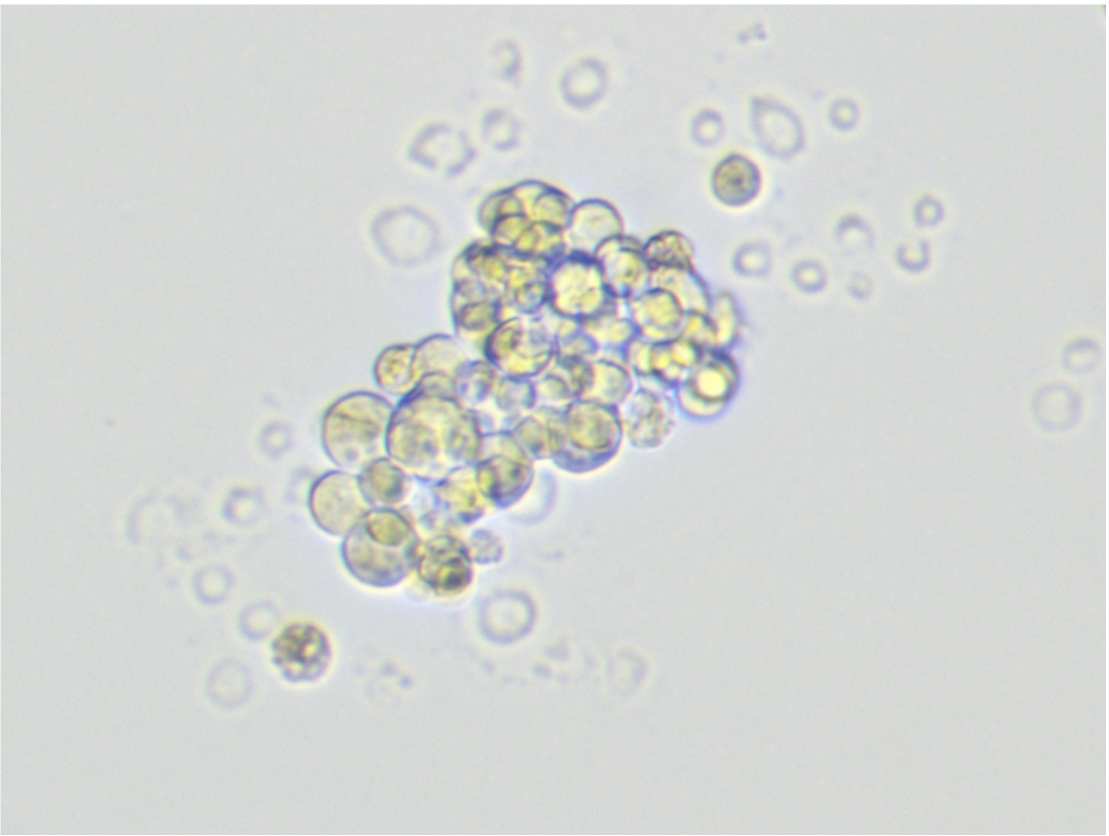

Figure 3A-Ishikawa Vitexin 5 µM

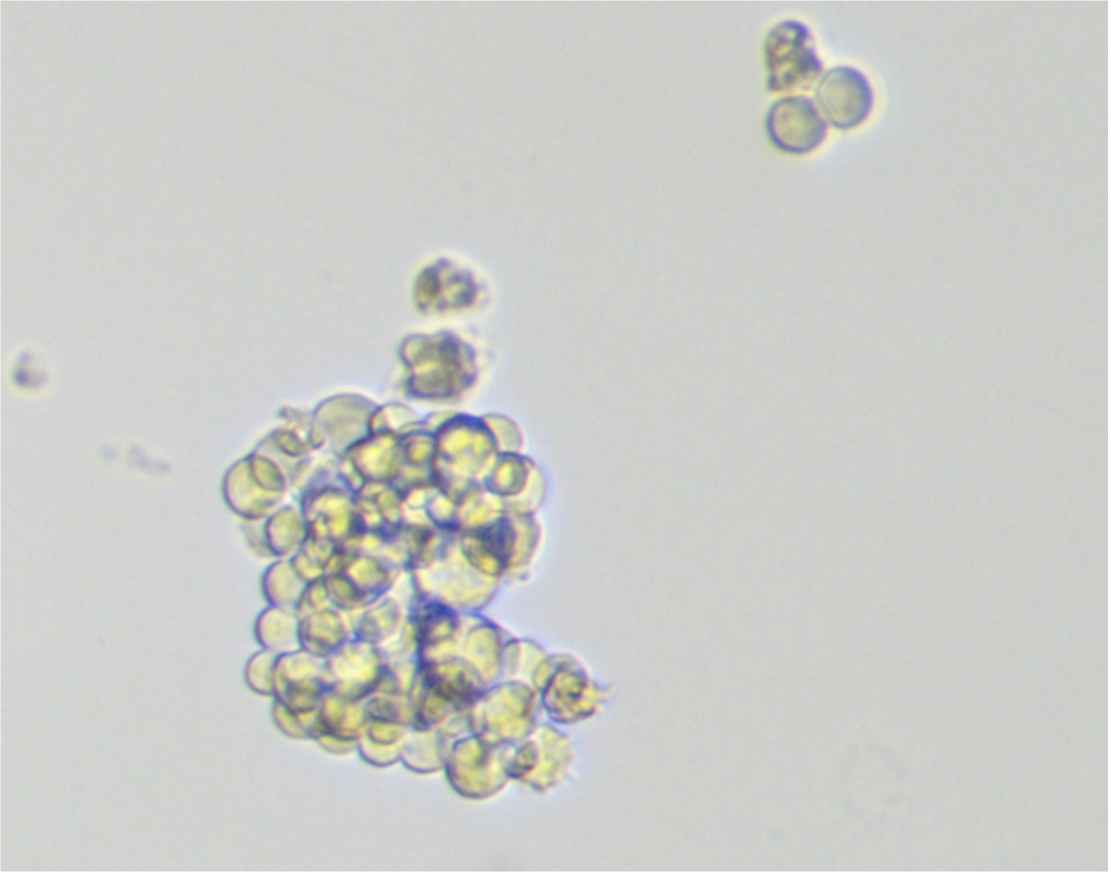

Figure 3A-Ishikawa Vitexin 10 µM

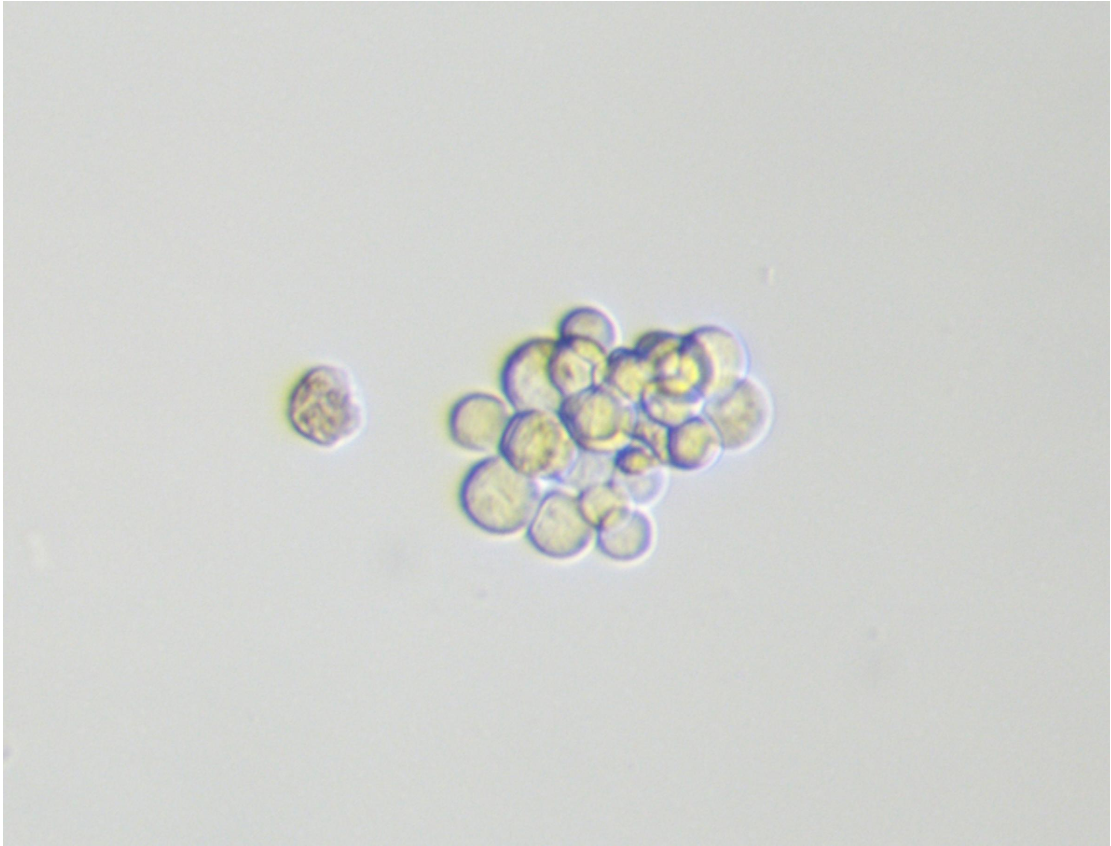

Figure 3A-Ishikawa Vitexin 0 µM

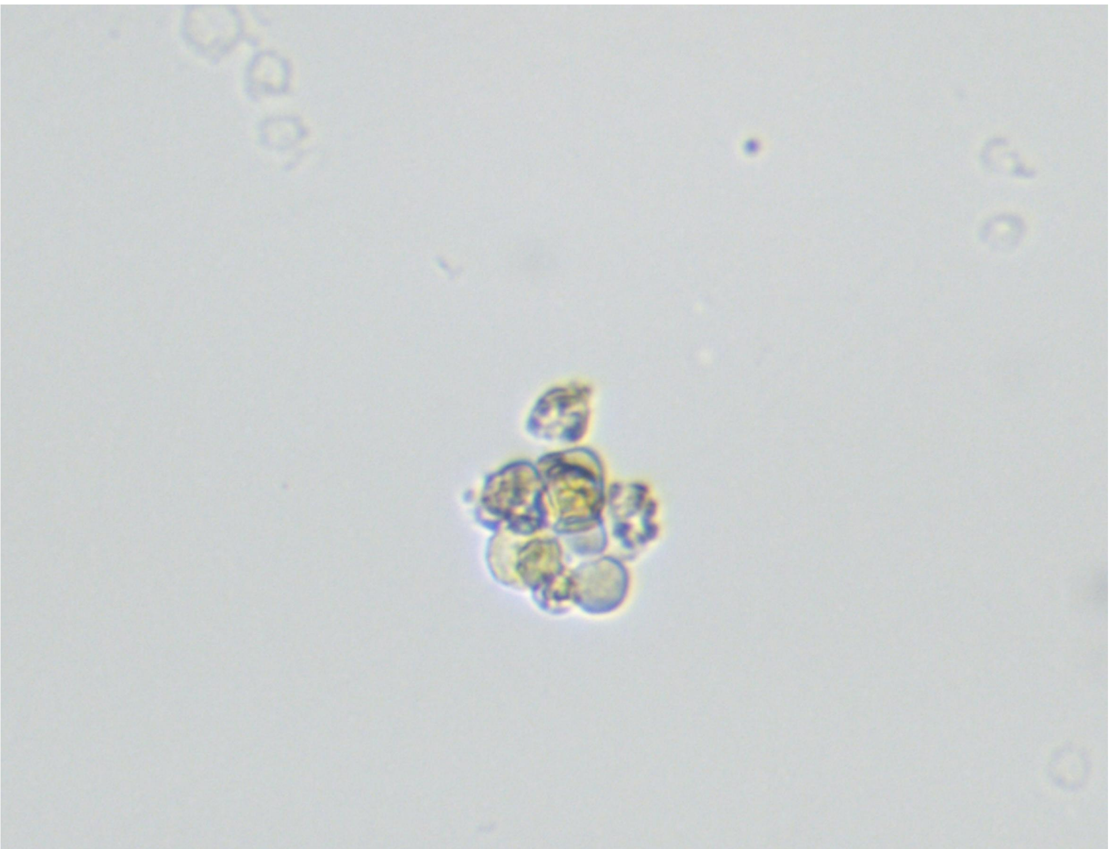

Figure 4E con

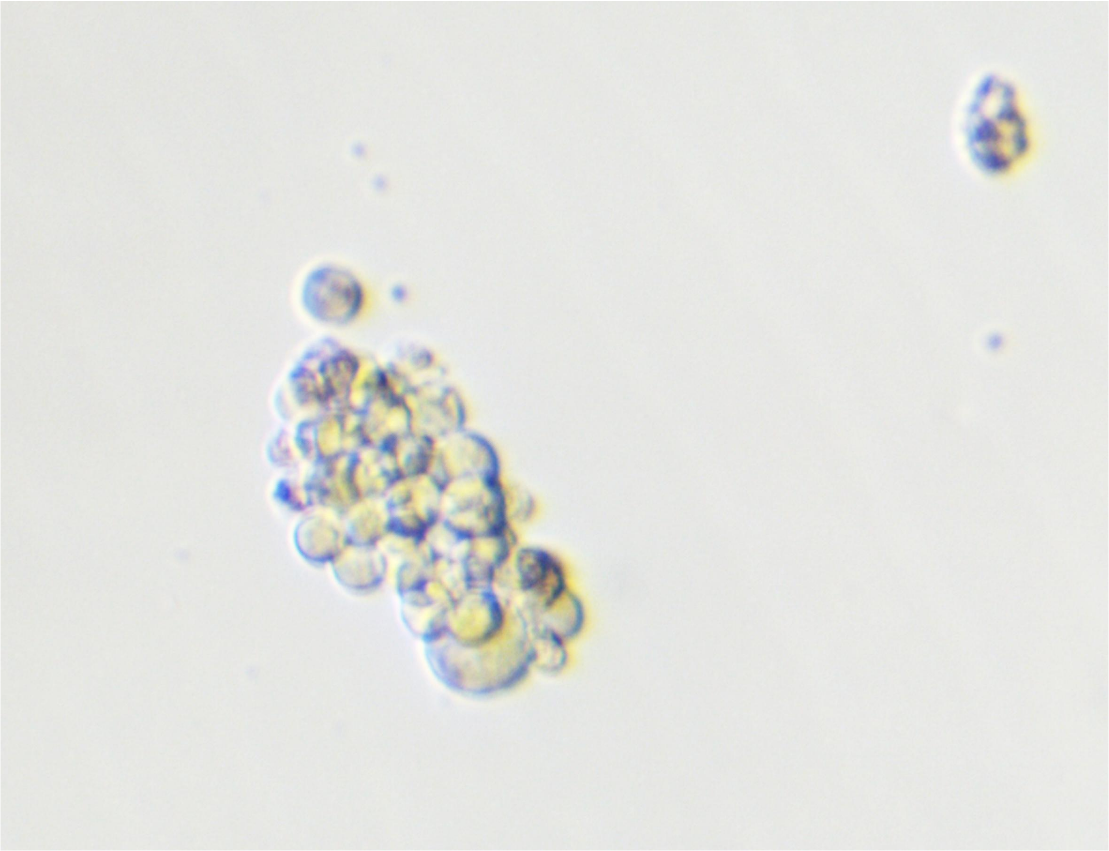

Figure 4E Vitexin 20 µM

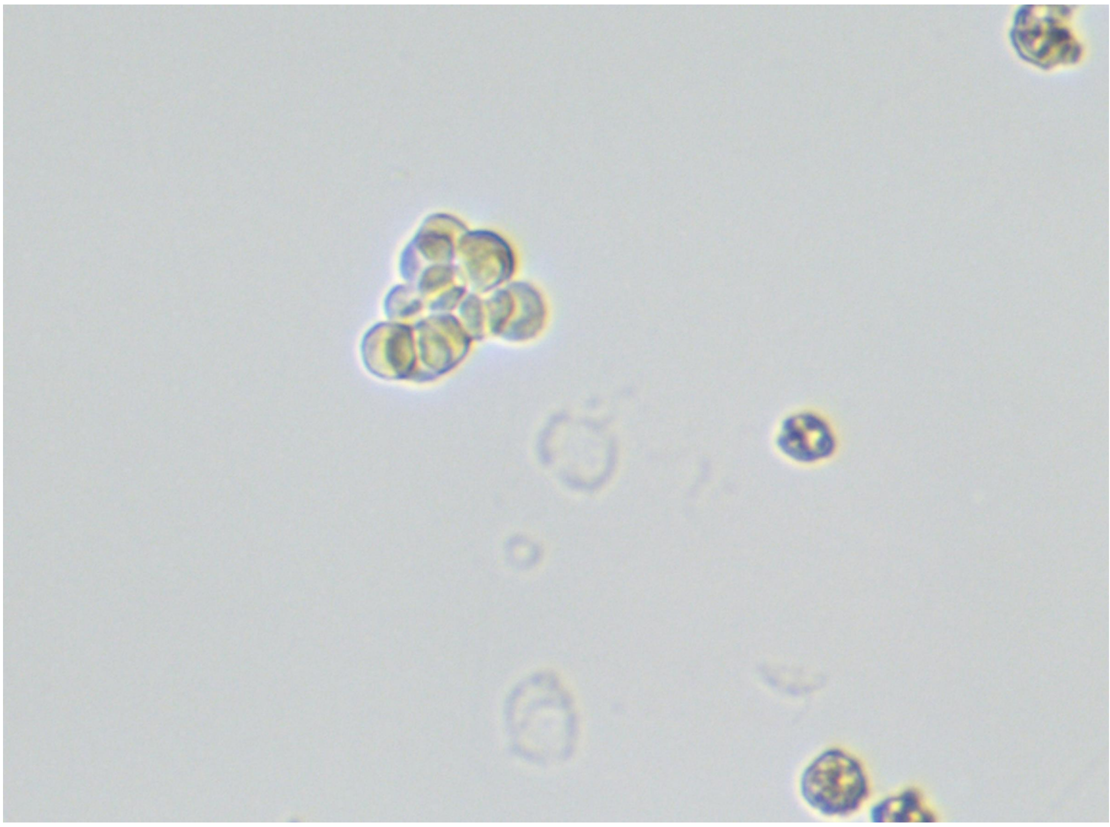

Figure 4E Vitexin 20 µM +740 Y-P

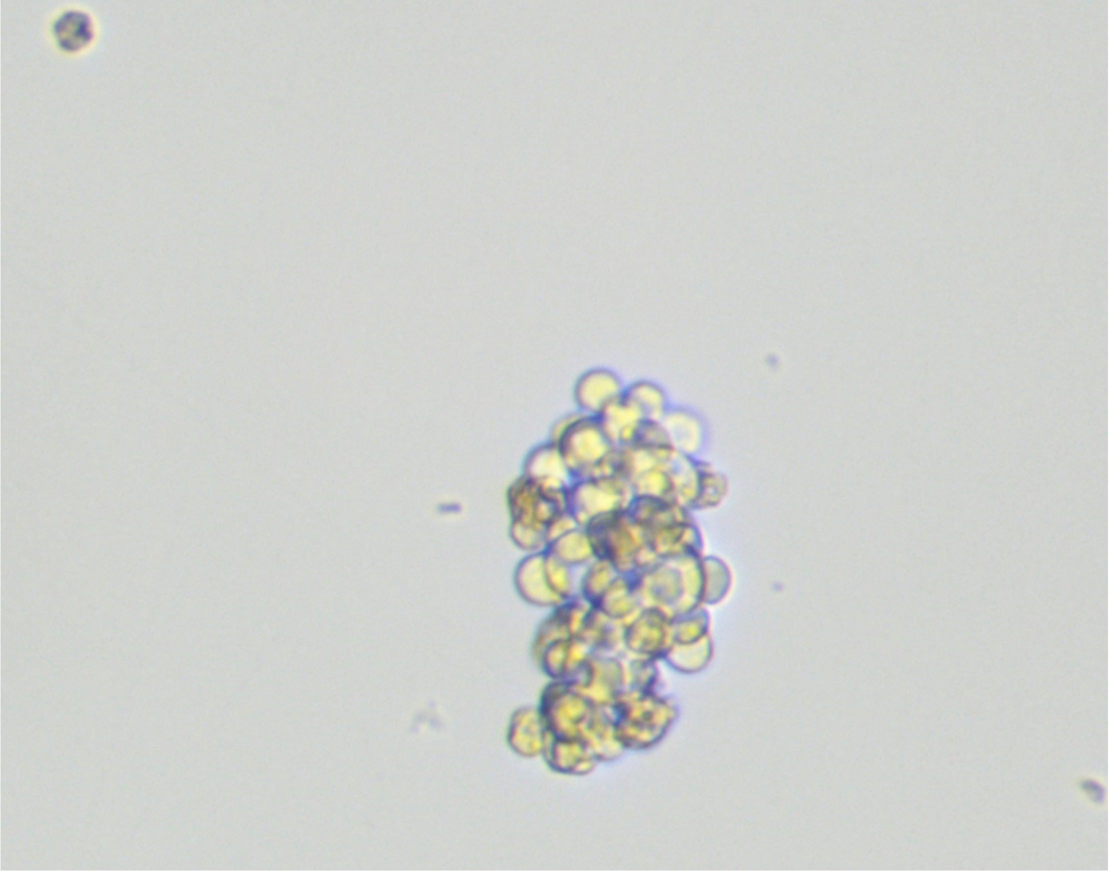

Supplement: Supplemental Material [file IPHB_A_2190774_SM2227.pdf]
